# Factors associated with suicide ideation among subway drivers in Korea

**DOI:** 10.1186/s40557-016-0120-5

**Published:** 2016-08-02

**Authors:** Junsu Byun, Hyoung-Ryoul Kim, Hye-Eun Lee, Se-Eun Kim, Jongin Lee

**Affiliations:** 1 Department of Occupational and Environmental Medicine, College of Medicine, The Catholic University of Korea, Seoul, Korea; 2 Healing Center, The Seoul Metropolitan Rapid Transit Cooperation, Seoul, Korea; 3 Cheongsong Health Center and County Hospital, Cheongsong, Korea

**Keywords:** Suicide, Suicide ideation, Subway drivers, Occupational stress

## Abstract

**Background:**

There were several suicide events of subway drivers in Korea. The aim of this study is to explore work-related factors associated with suicide ideation among subway drivers.

**Methods:**

We analyzed data from 980 male subway drivers. A section of the Korean version of the Composite International Diagnostic Interview (K-CIDI 2.1) was administered by trained interviewers to judge whether a driver has suicide ideation and to diagnose psychiatric disorders. A questionnaire was also administered to collect data on sociodemographic characteristics, work environments, occupational stress, person under train (PUT) experience, and work-related problems. Occupational stress was examined by using the Korean Occupational Stress Scale (KOSS). Logistic regression was applied to evaluate the association between work-related factors and suicide ideation among subway drivers.

**Results:**

Regarding work-related problems, conflict with passengers and sudden stops due to the emergency bell were significantly associated with suicide ideation. MDD, PTSD, and panic disorder were strongly associated with suicide ideation. In the analysis of occupational stress, insufficient job control (OR 2.34) and lack of reward (OR 2.52) were associated with suicide ideation even after being adjusted for psychiatric disorders and other work-related factors.

**Conclusions:**

Insufficient job control and lack of reward were associated with suicide ideation among subway drivers. Strategies for drivers to have autonomy while working and to achieve effort-reward balance should be implemented. Furthermore, drivers who have experienced negative work-related problems should be managed appropriately.

## Background

Subway drivers are one of the most vulnerable working groups for psychiatric injury or trauma [1]. Psychiatric problems may arise from the experience of an incident of death or severe injury during their driving [2]. The person under train (PUT) incident is a well-known negative experience encountered by drivers, and this is neither evitable nor preventable by a driver himself [3]. Many studies have shown a strong relationship between post-traumatic stress disorder (PTSD) symptoms and PUT experience in subway drivers [1, 3–5]. Besides PTSD symptoms and other psychiatric problems—depression, anxiety, and panic symptoms—are also prevalent among subway drivers [4, 6, 7].

This study has a background of suicide events among subway drivers who worked in a company in Seoul. Nine subway drivers have committed suicide since 2003. To evaluate preventable risk factors for suicide, a workers’ union and the Ministry of Labor recommended conducting an epidemiological survey. Therefore, an epidemiological survey was performed to examine whether PUT experience was related to suicide in subway drivers and to measure its effect on the mental health of subway drivers in 2007 [4]. Subsequently, the researchers made some recommendations for subway drivers who experienced PUT. One was installing platform screen doors, which have been installed in all subway stations run by the company. By installing them, the number of PUT events has decreased dramatically. However, after installing platform screen doors, suicide events of subway drivers still occurred. Especially, the consecutive deaths of drivers between 2012 and 2013 motivated this study. To investigate why subway drivers continue to commit suicide, an epidemiological survey was performed in 2013.

The survey consisted of several steps. First, we surveyed all the subway drivers of the company to collect data of basic demographic statistics, mental health status, and occupational environments. Drivers classified with suicide ideation or any psychiatric disorders were interviewed in depth or referred to psychiatry specialists for treatment. A qualitative study for interviewing some drivers was also done. By analyzing and assessing the relationship between mental health status of subway drivers and occupational environments, we proposed countermeasures to prevent subway drivers’ suicide. This study is a part of the survey; analysis of the mental health status of subway drivers and occupational factors, focusing on suicide ideation and contributing events. Information of other analyses in the same survey are available elsewhere [5]. The aim of this study is to investigate which work-related factors or stress could have effect on suicide ideation.

## Methods

### Study subjects

We examined subway drivers who were employed in a public subway company in Seoul. Of all the 998 drivers in the company, 995 participated in this study. We excluded data of 15 female drivers and analyzed data from only 980 male drivers because we expected gender difference of suicide ideation [8] and all cases of suicide were male. This study was approved by the Institutional Review Board of Seoul St. Mary’s Hospital (IRB No. KC13QNSI0168). All study subjects submitted informed consent and all the data were used after encryption.

### Survey questionnaire

All participants were requested to complete a questionnaire, which included questions on demographic information (age, working duration, level of education, and smoking status) and experiences of work-related problems, as well as the Korean Occupational Stress Scale (KOSS).

The KOSS was developed to measure occupational stress in the working population in Korea, and it consists of subscales of eight domains: physical environment, job demand, insufficient job control, interpersonal conflict, job insecurity, organizational system, lack of reward, and occupational climate. It represented high validity by high correlation with Job Content Questionnaire (JCQ), short form of Psychosocial Wellbeing Index (PWI-SF), and Mental Fatigue Scale (MFS) [9]. The KOSS yields scores from four-point Likert scales of each question by domains. We divided the participants into high- and low-occupational stress groups depending on those they scored above or below the median domain value. Cronbach’s alpha used to evaluate the internal reliability of the subscale was 0.512–0.822.

Demographical and socioeconomic characteristics of participants were investigated by the survey questionnaire; major questions included information about satisfaction with economic status, experience of visiting a medical doctor’s office, sleeping well, and exact sleeping time. The selected questions were the same with the previous study in 2007 [4]. To investigate work-related problems, we asked about experience of conflict with passengers, sudden stops due to the emergency bell, near accidents, breakdowns, and observing the PUT experience of colleagues within one year of their work. We also inquired their personal experience of PUT since they had started working.

### Suicide ideation

We used the Korean version of the Composite International Diagnostic Interview (K-CIDI 2.1) in the survey [10]. The CIDI is a fully standardized, structured interview designed to make psychiatric diagnosis using the Diagnostic and Statistical Manual of Mental Disorders 4^th^ edition (DSM-IV) [11, 12]. The K-CIDI was validated according to the World Health Organization guideline [13]. The drivers were structurally interviewed by well-trained nine nurses. Section S of the K-CIDI pertains to suicide ideation. Participants were assigned to the suicide ideation group (SIG) if they reported having seriously considered committing suicide in the past year. Others were assigned to the non-SIG (NSIG).

### Diagnosis of psychiatric disorders

The K-CIDI is designed to diagnose the 1-year and lifetime prevalence rates of psychiatric diseases, and is based on DSM-IV. All diagnoses were made by diagnostic questionnaires of K-CIDI. We obtained prevalence data for major depressive disorder (MDD), PTSD, general anxiety disorder (GAD), and panic disorder.

### Data analysis

All subjects were divided into two groups on the basis of suicide ideation. We used a chi-square test to show the differences of demographic characteristics, occupational experiences, and occupational stress between SIG and NSIG. Logistic regression was used to show the association by the prevalence of psychiatric disorders, the KOSS subscales, and work-related experiences.

We proposed two models. First, we calculated odd ratios (ORs) by each single variable of prevalence rates of psychiatric disorders, KOSS subscales, and work-related problems. Next we proposed the final model that contained all the variables we determined above to adjust confounding variables statistically.

All statistical analyses were performed using R 3.1.3.

## Result

Differences individual characteristics between the NSIG and SIG are shown in Table 1. The factors with the strongest differences were marital status and sleep. Divorced, separated, or bereaved drivers showed the proportion of suicide ideation the most highly while married drivers the opposite. The proportion of SIG was the lowest among those sleeping for 7 to 8 h (1.7 %) and reporting satisfying sleep (2.1 %). There was no difference in by working duration, level of education, smoking status, economic satisfaction, and experience of visiting a doctor’s office.

**Table 1:** Individual characteristics and suicide ideation

Variables		NSIG (%)	SIG (%)	*p*-value
		*N* = 947	*N* = 33	
Working duration (years)	<5	94 (97.9)	2 (2.1)	0.339
	5–10	183 (94.8)	10 (5.2)	
	10–20	559 (92.2)	16 (2.8)	
	≥20	111 (95.7)	5 (4.3)	
Level of education	High school	173 (97.2)	5 (2.8)	0.209
	Junior college	293 (95.1)	15 (4.9)	
	Above college	481 (97.4)	13 (2.6)	
Marital status	Unmarried	113 (94.2)	7 (5.8)	<0.001
	Married	815 (97.4)	22 (2.6)	
	Divorced/separated/bereaved	19 (82.6)	4 (17.4)	
Smoking status	Current smoker	306 (95.0)	16 (5.0)	0.150
	Former smoker	311 (97.5)	8 (2.5)	
	Never smoker	330 (97.3)	9 (2.7)	
Visiting a doctor’s office	Yes	455 (95.6)	20 (4.4)	0.214
	No	492 (97.4)	13 (2.6)	
Sleeping time (hours)	<7	404 (94.6)	22 (5.4)	0.020
	7–8	353 (98.3)	6 (1.7)	
	≥8	190 (97.4)	5 (2.6)	
Sleeping well	Yes	662 (97.9)	14 (2.1)	0.002
	No	285 (93.3)	19 (6.7)	
Economic satisfaction	Satisfactory	84 (97.7)	2 (2.3)	0.837
	Ordinary	461 (96.6)	16 (3.4)	
	Dissatisfactory	402 (96.4)	15 (3.6)	

*NSIG* non suicide ideation group, *SIG* suicide ideation group

Regarding work-related problems, experiences of conflict with passengers and sudden stops due to the emergency bell were significantly different between the two groups. In particular, neither one’s own nor a colleague’s PUT experience showed any difference between the two groups (Table 2).

**Table 2:** Experiences of work-related problems and suicide ideation

Variables		NSIG (%)	SIG (%)	*p*-value
		*N* = 947	*N* = 33	
Person under train (PUT)	Yes	302 (96.2)	12 (3.8)	0.725
	No	645 (96.8)	21 (3.2)	
Conflict with passengers	Yes	246 (94.3)	15 (5.7)	0.022
	No	701 (97.5)	18 (2.5)	
Sudden stop due to the emergency bell	Yes	600 (95.4)	29 (4.6)	0.007
	No	347 (98.9)	4 (1.1)	
Near accident	Yes	404 (95.7)	18 (4.3)	0.239
	No	543 (97.3)	15 (2.7)	
Breakdown	Yes	125 (99.2)	1 (0.8)	0.147
	No	822 (96.3)	32 (3.7)	
Colleague’s PUT experience	Yes	199 (94.8)	11 (5.2)	0.139
	No	748 (97.1)	22 (2.9)	

*NSIG* non-suicide ideation group, *SIG* suicide ideation group, *PUT* person under train

Most KOSS subscales except job insecurity, physical environment, and organizational system were significantly different between the two groups (Table 3).

**Table 3:** Occupational stress and suicide ideation

Variables	Score	NSIG (%)	SIG (%)	*p*-value
		*N* = 947	*N* = 33	
Physical environment	Low	481 (97.8)	11 (2.2)	0.073
	High	466 (95.5)	22 (4.5)	
Job demand	Low	588 (98.0)	12 (2.0)	0.005
	High	359 (94.5)	21 (5.5)	
Insufficient job control	Low	734 (97.6)	18 (2.4)	0.004
	High	213 (93.4)	15 (6.6)	
Interpersonal conflict	Low	378 (98.4)	6 (1.6)	0.020
	High	569 (95.5)	27 (4.5)	
Job insecurity	Low	63 (96.9)	2 (3.1)	1.000
	High	884 (96.6)	31 (3.4)	
Organizational system	Low	317 (97.8)	7 (2.2)	0.199
	High	630 (96.0)	26 (4.0)	
Lack of reward	Low	586 (98.2)	11 (1.8)	0.002
	High	361 (94.3)	22 (5.7)	
Occupational climate	Low	605 (97.7)	14 (2.3)	0.020
	High	342 (94.7)	19 (5.3)	

*NSIG* non suicide ideation group, *SIG* suicide ideation group

Psychiatric disorders were strongly associated with suicide ideation (Table 4). MDD, PTSD and panic disorder showed high ORs for suicide ideation. GAD also had a high OR for suicide ideation but it was not statistically significant. Both the 1-year and lifetime prevalence rates of the three psychiatric disorders (MDD, PTSD, and panic disorder) were associated with suicide ideation.

**Table 4:** Psychiatric disorders and suicide ideation: univariate logistic regression

Variables		NSIG (%)	SIG (%)	Odds ratio [95 % CI]
		*N* = 947	*N* = 33	
1 year prevalence	MDD	11 (1.2)	7 (21.2)	22.91 [7.89–63.14]
	PTSD	12 (1.3)	3 (9.1)	7.79 [1.71–26.11]
	Panic disorder	5 (0.5)	5 (15.2)	33.64 [8.90–127.53]
	General anxiety disorder	2 (0.2)	1 (3.0)	14.77 [0.68–158.03]
Lifetime prevalence	MDD	32 (3.4)	9 (27.3)	10.72 [4.42–24.30]
	PTSD	24 (2.5)	4 (12.1)	5.30 [1.49–14.85]
	Panic disorder	9 (1.0)	7 (21.2)	28.06 [9.40–81.28]
	General anxiety disorder	5 (0.5)	1 (3.0)	5.89 [0.30–37.92]

*NSIG* non suicide ideation group, *SIG* suicide ideation group, *MDD* major depressive disorder, *PTSD* post-traumatic stress disorder

The KOSS subscales and work-related problems were associated with suicide ideation (Table 5). In an unadjusted model, variables except job insecurity and organizational system showed significant ORs.

**Table 5:** Effect of occupational stress and work-related problems on suicide ideation

Scale	Unadjusted model	Adjusted model
	OR [95 % CI]	OR [95 % CI]
Physical environment	2.064 [1.011–4.465]	0.990 [0.436–2.318]
Job demand	2.866 [1.415–6.075]	1.973 [0.887–4.574]
Insufficient job control	2.872 [1.405–5.792]	2.215 [1.017–4.736]
Interpersonal conflict	2.989 [1.307–8.072]	1.710 [0.705–4.787]
Job insecurity	1.105 [0.324–6.919]	1.008 [0.253–7.137]
Organizational system	1.869 [0.836–4.714]	0.835 [0.351–2.263]
Lack of reward	3.247 [1.588–7.026]	2.236 [1.021–5.153]
Occupational climate	2.401 [1.195–4.939]	1.324 [0.597–3.094]
Conflict with passengers	2.375 [1.163–4.782]	1.647 [0.742–3.631]
Sudden stop due to the emergency bell	4.193 [1.634–14.226]	3.130 [1.113–11.353]

Adjusted model: adjustment for age, marital status, MDD, PTSD, GAD, panic disorder, and above variable

The variables of age, marital status, and prevalence rates of psychiatric disorders (MDD, PTSD, GAD and panic disorder) were added in the adjusted the model because they could act as confounding factors. In the adjusted model, insufficient job control and lack of reward were significantly associated with the suicide ideation. Sudden stop due to the emergency bell was also significantly associated.

## Discussion

Our study suggests that drivers having experiences of work-related problems, including conflict with passengers and sudden stops due to the emergency bell, tend to have suicide ideation. Occupational stress stemming from insufficient job control and lack of reward was also associated with suicide ideation. As mentioned in the introduction, many studies have focused on the relationship between PUT experience and psychiatric problems, mainly PTSD [1, 2, 4]. It is easy to assume that people who have experienced PUT incidents could have PTSD because the PUT experience could be highly traumatic. PTSD is associated with suicidal ideation and suicide attempts [14].

However, PTSD does not fully explain the series of suicides committed by subway drivers. Generally, PTSD has a course of onset within the first three months of trauma and lasts for months or years. Typically, individuals with PTSD are more likely to have symptoms of other mental disorders (depression, panic, etc.). However, in a previous study that investigated the same population in 2007, there was a negligible or weakly significant relationship between PUT experience and depressive symptoms [4]. Another study in France revealed that psycho-behavioral disorders observed in the immediate aftermath of PUT accidents disappeared within a year [15].

Because the model explaining that suicide occurs because of PTSD after PUT accidents is incomplete, and the completion of installation of the platform screen door system in the Seoul Subway did not have the desired effects on the suicide rate of subway drivers, it is necessary to determine other factors affecting psychiatric morbidity and suicide in subway drivers. Work-related factors such as sudden stops from the emergency bell and near accidents, rather than PUT experience, had strong relationships with depressive symptoms in the 2007 study [6]. As the 2007 study did not assess suicide ideation, our results cannot be compared with those directly.

Events that were related to suicide ideation in our study—conflict with passengers and sudden stops due to the emergency bell—should be managed as major risk factors for suicide. These two risk factors have a common feature—they are related to passengers—so the stressors might persist while the other work-related factors we investigated are one-time events. These two experiences might impose an emotional demand that can lead to suicide ideation [16].

As we described before, the epidemiological survey contained qualitative interviews of sampled drivers. According to the interview content, a driver should have stopped his train urgently because a passenger’s bag was caught in the car door. The passenger blamed the driver continuously, so he compensated the passenger for the bag out of his own pocket. Furthermore, his experience was publicly shared with other drivers as a bad example of work performance in a cluster education session. He confided that he experienced suicide ideation owing to this event and its consequences. This experience is a kind of emotional trauma that could induce psychiatric disorders.

In the same context, occupational stress would be more stressful to workers than a one-time event like a PUT accident because the former tends to be chronic and consistent. Many theories explain the fundamental causes of occupational stress. The KOSS model consists of eight domains. In our study, insufficient job control and lack of reward were two domains that affected suicide ideation while the other six were not significantly related to suicide ideation after we adjusted experiences of work-related problems and psychiatric disorders. This result is inconsistent with findings from other workers who are vulnerable to occupational stress. In studies on occupational stress in firefighters, KOSS domains were linked to depressive symptoms [17, 18] and sleep disorders [19]. However, the domains with significant associations differed from those in our study, perhaps because of differences in the characteristics of workers and related outcomes. In a study comparing subway workers with firefighters, scores of job insecurity, organizational system, and lack of reward among subway workers were better than those of firefighters [20].

Psychiatric disorders explain the pathway from occupational stress to suicide ideation. While GAD did not show any significant relationship with suicide ideation, the associations were strong for MDD, PTSD, and panic disorder. After we adjusted for these disorders in the logistic regression model, the effects of insufficient job control and lack of reward on suicide ideation were still significant while the impact of physical environment, job demand, interpersonal conflict, and occupational climate disappeared. This result implies that most occupational stress domains affect suicide ideation via psychiatric disorders, including MDD, PTSD, and panic disorder. However, insufficient job control and lack of reward directly affect suicide ideation.

Subway drivers suffered work-related problems in the aspect of insufficient control, which pertains to irregular working conditions, irregular meal times, and burden related to on-time transit service with prolonged immobilization. To reduce suicide rates, organizational and systematic approaches to assure sufficient control are needed. Further, interventions are necessary for drivers who experience conflict with passengers or sudden stops due to the emergency bell.

This study has some limitations. We used cross-sectional data and therefore could not make definite conclusions about causal or temporal relationships. People who have suicide ideation might feel greater occupational stress than others. However, it is more rational to consider that experiences of work-related problems and stress precede suicide ideation because development of suicide ideation requires sources of pain that decrease the desire to live [21].

We did not measure attempted or completed suicides directly. However, some previous studies support our methodology of measuring suicide ideation. Suicide ideation is followed by suicide event [21, 22]. Many studies have revealed that suicide ideation or consideration on death was related with suicide attempts, as well as the exact prevalence suicide events [23–25]. Although most patients with suicide ideation do not commit suicide ultimately, a management against suicide ideation may preclude more serious suicide attempts or events [26].

We consider that very high participation rate (99.7 %) and using evaluating tools with good validity such as K-CIDI were the strength of this study.

While we stated that sufficient control and reward are the factors most strongly associated with suicide ideation among subway drivers, we also pointed out that this trend might differ according to working groups. Countermeasures are needed to subway protect drivers who have experienced conflict with passengers and sudden stops due to the emergency bell. Furthermore, policies that ensure job control would be effective in lowering the suicide rate of subway drivers. We expect that the methods we used can be applied to other workers who experience persistent occupational stress, to identify weak points and solutions.

## Conclusion

Insufficient job control and lack of reward were associated with suicide ideation among subway drivers. Strategies for drivers to have autonomy while working and to achieve effort-reward balance should be implemented. Further, drivers who have experienced negative work-related problems, especially conflict with passengers and/or sudden stop due to the emergency bell should be managed appropriately.
